# Gender and life-stage dependent reactions to the risk of radioactive contamination: A survey experiment in Sweden

**DOI:** 10.1371/journal.pone.0232259

**Published:** 2020-04-30

**Authors:** Joel Rasmussen, Jens Ewald, Thomas Sterner

**Affiliations:** 1 Media and Communication Studies, School of Humanities, Education and Social Sciences, Örebro University, Örebro, Sweden; 2 Department of Economics, School of Business, Economics and Law, Gothenburg University, Gothenburg, Sweden; Fukushima Medical University School of Medicine, JAPAN

## Abstract

This article proposes and examines gender and life-stage factors as determinants of public worry and risk avoidance in a nuclear fallout scenario. Drawing on a survey (N 2,291) conducted in Sweden, the article demonstrates statistically significant results that women as well as parents with children at home are more likely to express high levels of worry for radiation exposure and have a preference to move away from a fallout area despite assurance of successful remediation. Moreover, a negative relationship is shown between age and both worry for radiation exposure and preference to move. These novel results from Northern Europe thus support a life-stage framing of public risk attitudes. As radiation physicists develop new methods showing that women and children are at higher risk of cancer than other groups at the same radiation exposure, we may actually see the precaution among women and parents as a regulating mechanism for the higher objective risk they face. The results are moreover in agreement with studies of public risk reactions in Japan, creating a strong knowledge base that human-induced radiation pollution is largely an intolerable risk to the public. Considering the public opinion, managing an intolerable risk through risk mitigation by remediation alone is likely insufficient in many cases. A viable strategy would offer a range of social support options that enable individual decision-making and the protection of risk groups.

## Introduction

A large nuclear accident presents challenges of an unusual magnitude to decision-makers. The radiation effects are always worst initially and all decisions will require skilled knowledge that is quite scarce. There will be an urgent need for large-scale action and it is thus important to develop plans and analyses long before an emergency happens. Decision-making on the conditions of citizens’ relocation and possible return in the event of a nuclear accident with radioactive fallout poses some special challenges. While the consensus of governing bodies on the management of radioactive fallout in populated areas is indeed to focus on relocation, decontamination, and citizens’ eventual return [1], accidents that have occurred suggest that a large proportion of evacuees are reluctant to follow government advice and move back. For instance, the nuclear accident at the Fukushima Daiichi power station in Japan in March, 2011, saw 165,000 citizens relocating in a couple of months [2, 3] and 234,000 by the end of the same year [4]. Determined to make all areas habitable again, the Japanese government invested immensely in recovery, amounting to 25 trillion yen up until 2016, or about 25% of their regular, annual state budget [3]. The government also endorsed citizens’ return by raising the permitted annual exposure to ionizing radiation from 1 mSv (millisievert) to 20 mSv, and by gradually ending the facilitation and subsidies of temporary housing [5]. Yet, survey results from two towns within the Fukushima prefecture demonstrate that only 30% of the evacuees were willing to return a year after the accident. Furthermore, the number of evacuees who refuse to return increases with time [6]. Four and a half years after the accident, population estimates from the Fukushima Prefecture show that less than half had returned. A month after the ban on returning had been lifted from one of its towns, Naraha, in 2015, only one in seven followed official advice and moved back [7]. Researchers cite citizens’ concerns about the health risks of exposure to ionizing radiation as the main reason why many do not return [6, 8]. A particularly low percentage of young adults, women and children return [9].

Japan’s example of differences in risk perception and behavior is one of many that the extensive field of risk research has presented. In recurring studies, certain demographics and life situations entail an inclination to perceive risk as higher than others do and favor risk avoidance [10, 11]. Providing grounds for concern and precaution, the lifetime attributable risk (LAR) of cancer for newborn girls is 5.4% when ground deposition of Cs-137 is 1.0 MBq/m^2^ (i.e., a contamination level below the approximate 1.5 MBq/m^2^ limit set for mandatory evacuation after both the Chernobyl and the Fukushima Daiichi accidents). LAR of cancer for newborn girls compared to 30-year-old women, and 30-year-old women compared to men, is higher by a factor of 4.5 and 1.2 respectively [12]. This suggests that women who are (objectively) both more vulnerable themselves and affected by a newborn would be quite right in being more concerned about radiation risk following a nuclear accident. The same would apply to parents of young children. Differences in risk perception are moreover problematic to change through knowledge-sharing or persuasion [13]. When not understood nor responded to properly, they often lead to prolonged conflicts with great human and financial costs [14]. Research has thus suggested that societies’ preparedness for nuclear accidents can be improved if governing bodies gain a better understanding of how citizens’ risk perception and behavior vary by demographics [8, 15]. Dupont [16] suggests that two such factors would be gender and parenthood status. This corresponds to Morita et al.’s [8] findings regarding voluntary evacuation in Japan, which, in addition to gender and household type, found age to be a key factor. Whether the same would apply in another part of the world is not certain. For instance, Olofsson and Rashid [17] contend that varying degrees of social security and status explain variation in risk perception. Their results indicate that, in a country like Sweden with far-reaching gender equality, there are no significant gender differences in risk perception.

The varying results from these studies and the suggested importance of demographics in some of them prompt our research on the meaning of gender and life-stage factors in a nuclear fallout scenario. It seems likely that in addition to gender, different life stages imply variations in risk response, meaning that factors related to different phases of the human life trajectory are distinguishable [18] and have significance for how risk is viewed and addressed. Research suggests that mechanisms causing correlation between demographics and risk response extend both our psychological precaution system developed through human evolution [19, 20] and the socio-cultural environment [21, 22]. Our research thus aims to study how gender and life-stage factors including age group and having home-based children relate to levels of worry for radiation exposure and preference for radiation risk avoidance. By worry, we refer to “negatively affect-laden and relatively uncontrollable” thoughts and processes in the face of risk [23] whereas levels of risk avoidance refer to the extent to which people are prone to select “a path that does not touch on the risk.” [24] To accomplish this, the study analyzes the results of a survey containing a scenario description and questions answered by 2,291 Swedish citizens.

The study’s contribution will be two-fold. First, a body of knowledge on risk perception and behavior in relation to nuclear accidents and radiation has accumulated lately with several studies from Japan [8, 9] implying that different geographically-focused research is needed to understand risk responses globally, to which the present study conducted in Northern Europe is an addition. Secondly, our results may be useful for future risk management. Because risk management is about preventing shock and crisis in society using methods that are appropriate depending on whether the public considers a risk acceptable, tolerable, or intolerable [24], our findings on levels of worry and preference for risk avoidance can usefully indicate how tolerable a recovery area is perceived to be. A risk mitigation measure that is adequate when the population sees the risk as tolerable is likely to be inadequate if large sections of the population perceive the risk as intolerable. Returning to a place will be less attractive if only some people return and these mechanisms may affect the choice between sanitizing an area and evacuating it more permanently.

In the following, we address research on group differences in risk perceptions which, theme by theme, leads to the presentation of the study’s hypotheses. Thereafter, the study’s method and analysis are presented. We conclude by discussing the results in relation to previous research.

### Gender effects

Recently, research comparing men’s and women’s risk perception has demonstrated surprising similarities and no statistically significant differences [17]. Concerns about technological systems proved to be the variable showing least difference. Pointing to the correlation between far-reaching social equality and high degree of similarity in risk perception, the researchers suggest a social explanatory model for risk perception. The results certainly prompt further studies as the findings deviate from an otherwise pervasive trend.

For quite some time, a large body of research has found that women express both a higher level of worry for risks and preference for risk avoidance than men do [10, 25, 26]. Indicating greater risk proneness, men score higher on most of the factors that public health research selects for testing, such as smoking [27], excessive alcohol consumption [28], and drinking and driving [29]. Arguably, men also take greater health risks in working life than women do, as more of them are exposed to physical and chemical hazards at work [30, 31]. The top risks that cause men to lose life-years are more often of a behavioral nature than the risks that have the greatest impact on women’s longevity [30, 32]. In addition, men are less likely than women to seek medical treatment and when they do, they do not speak as often and openly about their symptoms [33] and yet they report better subjective health [34].

Continuing with research on technological and environmental risks, among 25 risks measured, the third and fourth largest differences between the sexes were found regarding risks associated with radioactive waste and nuclear power plants [14]. In another study, examining 13 risks to the individual, nuclear power plants and radioactive waste were again among the top four items showing the greatest difference between the sexes [35]. Across ethnic groups, men show less concern than women do that exposure to chemical and radioactive substances would affect people’s health negatively. For instance, a question that is judged to entail significantly lower risk by men than by women is if roads for transport of hazardous chemicals and radioactive materials located near homes and farms would adversely affect house prices and consumer acceptance of farm products [14]. A study conducted after the Chernobyl accident demonstrates that women consistently express perceptions of higher risk regarding nuclear power and related radiation risks than men, and more behavioral change when exposed to risks including contaminated food [36].

Finally, research in the aftermath of the severe reactor accident in Fukushima Daiichi found more men endorsing staying in areas affected by radioactive fallout than women. This meant that several families split their living space between two locations–the men staying in areas of increased radiation risk if they could continue to work there, while women and children moved elsewhere. Another dominant feature of women’s post-accident risk perception and behavior was caution regarding the choice of food [21]. In public, women’s perception of high risk and precautious choices were portrayed as inferior, though, as if unfounded and caused by harmful rumors (fūhyōhigai). Despite the scientific uncertainty regarding the long term effects of low-dose ionizing radiation on children [37], the media and experts accused these women of lacking scientific know-how and being hysterical over radiation, sometimes calling them “radiation brain moms” [38]. New research indeed demonstrates higher LAR of cancer for women than for men at exposure to the same ground deposition level of Cs-137 [12], thus providing grounds for concern among women.

Causing several questions to remain open, though, a study [17] conducted in Northern Europe declared that “there is no significant difference between men and women in risk perception.” Since this study did indeed include risks from technological systems but not nuclear accidents nor ionizing radiation per se, there are reasons to base our hypotheses on the evidence available from the majority of studies showing that gender differences in perception of ionizing radiation have been particularly large. We therefore pose the following hypotheses:

*Hypothesis 1*. *Women express higher levels of worry for the risk of ionizing radiation exposure than men do*.
*Hypothesis 2*. *Women more likely prefer to avoid ionizing radiation risk by moving than men do*.

### Age effects

Citizens’ estimations of risk and patterns of behavioral risk precaution have been found to vary with age. These results seem to differ for different risk domains though. The oldest portion of the population is less worried than the younger population for known risks, associated with traffic or recreational activities, but more than others they reduce and avoid these risks [39] and typically demand that they are mitigated through public funding [40]. Risk-taking in everyday life thus appears to decrease linearly with age, indicating the increasing risk of health problems at an older age and awareness thereof [19, 41].

After the 2011 Fukushima nuclear disaster, results from surveys are mixed and not entirely conclusive. Hidaka et al. [42] found that, among decontamination workers, participants aged 61 years and more demonstrated higher degrees of worry over ionizing radiation. Moreover, studying evacuees from the accident, Suzuki et al. [43] demonstrate that older citizens (65 years and older) show greater concern than younger groups for the immediate effects of ionizing radiation, but results are the reverse for concerns regarding long-term and genetic health effects. Since concerns about ionizing radiation correlate strongly with people’s decision to relocate [6], and more young citizens move from contaminated areas than older people do [9], it seems consistent that Fukusawa et al. [44] found significant correlation between younger groups (compared to those aged 65 years and older) and worry over ionizing radiation.

As for post-accident behavior, Zhang et al. [9] found, three years after the accident, that few children and adult women returned after decontamination (e.g, 35% of children), but that elderly people returned to a high extent (86%). Moreover, those living with elderly relatives (70 years or older) were somewhat more likely (odds ratio 1.18) to remain in a pollution-affected area than those who did not [8]. Reasons stated for older people to stay are that the health risks of ionizing radiation are relatively low for them (compared to children and adolescents), that they more often than others have nostalgic feelings for their place of residence and, in tandem with gradually weakening health, and consider it economically and practically difficult to relocate and change lifestyle [9]. In light of these results, the following hypotheses were formulated:

*Hypothesis 3*. *Younger respondents express higher levels of worry for the risk of radiation exposure than the older ones do*.
*Hypothesis 4*. *Younger respondents more likely prefer to avoid ionizing radiation risk by moving than older respondents do*.

### Parental effects

In addition to gender and age, having children or not appears to be a demographic variable that should interest risk management. Research into actual crises, as well as experimental and scenario-based studies, shows that parents who have children living at home express perceptions of higher risk and avoid risk to a greater extent than others do [20, 36, 45–49]. After several parts of Europe had been affected by nuclear fallout from the Chernobyl accident, Drottz Sjöberg [36] found that parents consistently expressed perception of higher risk, more worry and behavioral change than non-parents, and this applied to all geographical locations, with varying degrees of radioactive deposition, where the study was conducted. Also in other areas than radiation, Wang et al. [20] demonstrate that parents compared to non-parents have a higher propensity to avoid behaviors that pose a health risk. In their study of what factors influence risk avoidance, Eibach and Mock [45] finds that it is the parental role that is crucial and not a personal trait of people who are positive to having children. When their parenting role is salient, people perceive higher risk and more likely choose to avoid risk than when the role is not salient or when compared to non-parents. Furthermore, a study on adults’ willingness to pay (WTP) for their own health risk reduction, finds that WTP is influenced by parenthood status, how many children in a particular age span they have, and whether the child or children are still living in the household [46]. This indicates that parents with children living at home are prone to sacrifice more for safety in a risky situation, which may also apply to behavioral attitudes in our study’s scenario of increased ionizing radiation risk, something that Drottz Sjöberg [36] at least indicated. Also, a WTP study, Liu et al. [47], shows that if a risk entails longer disease time or seriousness, then mothers are prepared to avoid it by expressing higher WTP. This suggests that the propensity among parents to avoid ionizing radiation, which may affect several generations, would be very high. Furthermore, the altruism of parents’ risk avoidance is evident in a study finding that the average mother’s WTP to avoid a mild illness is higher for her child than for herself [47] and in a study demonstrating that both parents express much higher WTP for their children than for themselves or other adults when life-threatening illness is that stake [48]. Yet another study demonstrates that parents’ WTP is about twice as high for their children than for themselves, although WTP decreases with high fertility, and the difference in WTP for adults and children is leveled out as the children reach adulthood [49]. In light of the above research results, but also because thirty years have passed since Drottz Sjöberg’s study [36] and perceptions may differ regarding different risks and scenarios, we test the following hypotheses:

*Hypothesis 5*. *Parents who have home-based children express higher levels of worry for the risk of radiation exposure than others do*.
*Hypothesis 6*. *Parents who have children in the home more likely prefer to avoid ionizing radiation risk by moving than others do*.

## Materials and methods

### Survey design

We carried out a survey in the period 22 February 2019 to 28 March 2019. The survey was sent out by e-mail as a part of the Swedish citizen panel by the Laboratory of Opinion Research (LORE) at the University of Gothenburg. 3,800 adult Swedish citizens were invited to participate in the study, from which 2,291 participated in the survey (a maximum of three reminders was sent out). Out of those, 1,068 are from a national probability-based sample stratified on gender, age, and education. The remaining respondents are targeted residents of any of the three Swedish municipalities with operational nuclear power plants, namely Halland (446 respondents), Kalmar (204 respondents), and Uppsala (573 respondents). The sampling of these respondents was stratified on gender. This sampling strategy enables the current study to examine gender differences but also estimate the importance of proximity to nuclear power plants. Sample errors consisted of some instances of missing data. Between 2% and 6% of the units lacked a response to some of the questions studied and were excluded. The significance level was set at P < 0.05. The study complies with the ethical standards for research involving human participants. Since consent was given as the questionnaire was voluntarily submitted, the data is anonymized, and because the survey responses are not of a sensitive, personal nature, no ethical examination was needed.

Before answering the survey questions, we presented the respondents with the hypothetical scenario in which a nuclear accident has occurred in Sweden and the respondent’s neighborhood has been affected by nuclear fallout and undergone remediation. The scenario description is available in the S1 Appendix and the raw data in the S1 Raw data. We dropped all answers from respondents spending less than five seconds reading the instructions (46 respondents).

### Response variables

To measure worry concerning radiation exposure, we asked the respondents: “To what extent would you feel worried over radioactive substances in your home, despite the fact that measurements show that the radiation levels are harmless? The answer choices were: “To a very small extent”, “To a somewhat small extent”, “Neither small nor large extent”, “To a somewhat large extent”, and “To a very large extent”.

To determine whether respondents prefer managing the risk of radiation exposure through risk avoidance, we asked: “How likely is it that you would continue to live in your home after it has been declared safe by the authorities?”. The answer choices provided were: “Very likely”, “Somewhat likely”, “Not very likely”, “Not at all likely”.

### Gender and life-stage variables

Our variables of interest are family situation, gender, and age group. In regards to family situation, we asked the respondents if they live or regularly live with one or more children. When asking for gender we gave the alternatives “Female”, “Male” and “Other”. No respondent self-identified as “Other”. We asked the respondents for their age and categorized them in the age groups “Less than 40 years”, “40 to 59 years”, “60 years and above”.

### Control variables

Control variables used in our multivariate statistical models include sample group, proximity to nearest nuclear power plant, income, educational level and membership of any local organization. The sample groups are Halland, Kalmar, Uppsala, and national. The proximity to the nearest power plant is represented by the answers to the question “How far is it from your home to the nearest power plant?” with the response alternatives “Less than 20 kilometers”, “20–50 kilometers”, “50–100 kilometers”, “More than 100 km”, and “Do not know”. The information is included as dummies in the models.

### Empirical strategy

We perform both non-parametric testing and regression testing. We conduct non-parametric testing to identify the overall relationship between our response variables and variables of interest without assuming any underlying distribution of the data. We calculate the mean score of our response variables with the corresponding 95% confidence intervals (95% CIs) over each of our variables of interest. We perform Mann–Whitney U-testing to identify differences between groups, which do not require any assumptions on the distribution of the data. We also perform multivariate regression analysis to identify the effect of the variables of interest while holding other possible cofounding factors constant. In this analysis, we use the response variables as dependent variables and different combinations of the variables of interest and control variables as regressors. Since the response variables are ordinal, we utilize the ordered logit model to estimate the outcome effects. For all statistical analysis, we used the statistical software Stata 16.1.

We present three regression model specifications. Model 1 comprises univariate models where we include the variables of interest as regressors separately. Model 2 includes the variables of interest separately together with all control variables. Model 3 includes all variables of interest and control variables as regressors simultaneously. We present the coefficients as adjusted odds ratios (aORs).

## Results

Counts and ratios of respondents in each group are presented in Table 1 together with the response variables. For detailed descriptive statistics of all variables, see S1–S4 Tables.

**Table 1:** Respondents by response variables and variables of interest.

Response variables	N	%
*Worry for radiation exposure*		
To a very small extent	132	6.0
To a somewhat small extent	444	20.2
Neither small nor large extent	348	15.8
To a somewhat large extent	830	37.8
To a very large extent	444	20.2
Total	2198	100
*Likeliness to stay in a decontaminated area*		
Very likely	218	10.0
Somewhat likely	732	33.5
Not very likely	890	40.7
Not at all likely	345	15.8
Total	2185	100
**Variables of interest**	** **	** **
*Family situation*		
No child	1552	72.3
≥1 child	596	27.8
Total	2148	100
*Gender*		
Male	1163	51.8
Female	1082	48.2
Total	2245	100
*Age*		
< 40 years	543	25.4
40–59 years	762	35.6
≥60 years	837	39.1
Total	2142	100

### Non-parametric analysis

**Fig 1:**
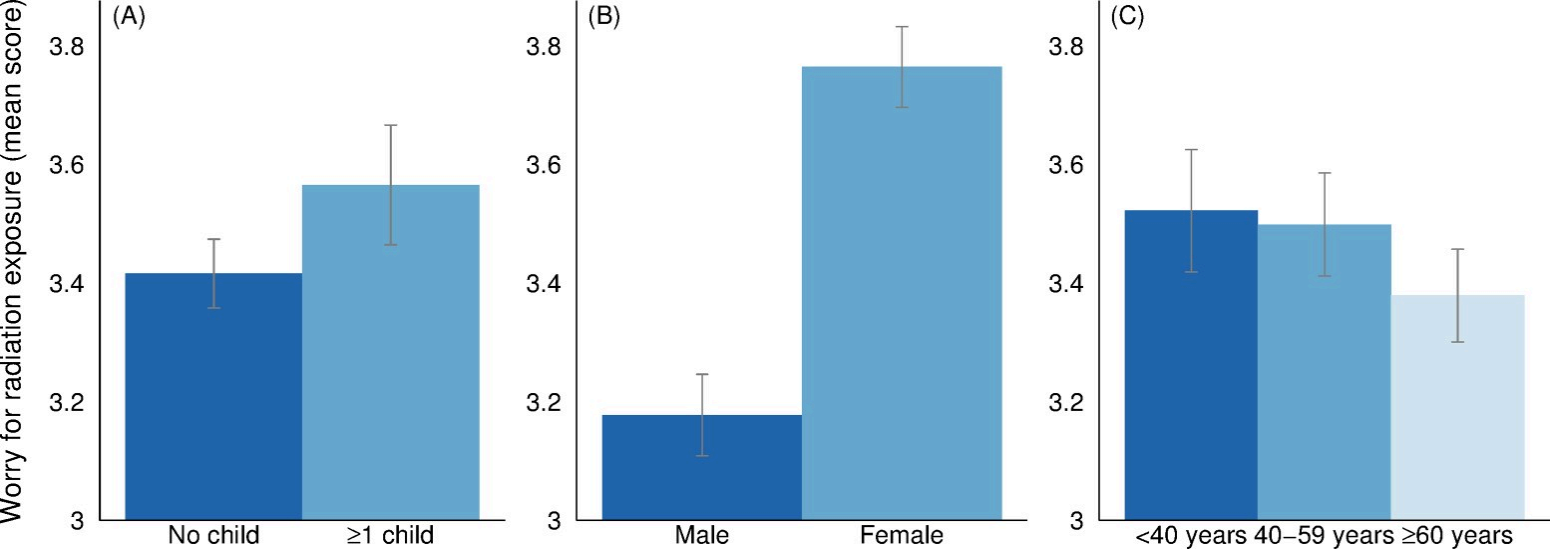
Worry for radiation exposure by family situation, gender, and age. Confidence intervals are displayed at the 95% level.

Fig 1 displays the mean score of worry for radiation exposure by family situation, gender, and age. Peoples’ family situation appears to matter for their level of worry for radiation exposure. In the five-point Likert scale, respondents without any children report a mean score for worry of 3.42 (the 95% confidence interval is 3.36–3.47) while those with at least one child in their household report on average a score of 3.57 (3.46–3.67). To compare the two groups we perform the non-parametric Mann-Whitley (MW) test and it reports the p-value 0.001. Hence, the family-situation difference is statistically significant. We also find gender to be an important determinant of worry for radiation exposure. Female respondents report an average score of 3.76 while male respondents report on average a score of 3.18 and the difference is highly statistically significant (MW p-value = 0.000). In regards to age, a negative relationship between age and worry for radiation exposure can be seen. However, there are no statistically significant differences between the two youngest age groups (respondents under 40 years old and between 40 and 59 years old). Respondents over 60 years old on the other hand appear to experience significantly less worry than the other two age groups (MW p-value = 0.015). Furthermore, Pearson’s chi-squared test gives an overall p-value of 0.008 –indicating that there is an overall negative relationship between age and worry for radiation exposure.

**Fig 2:**
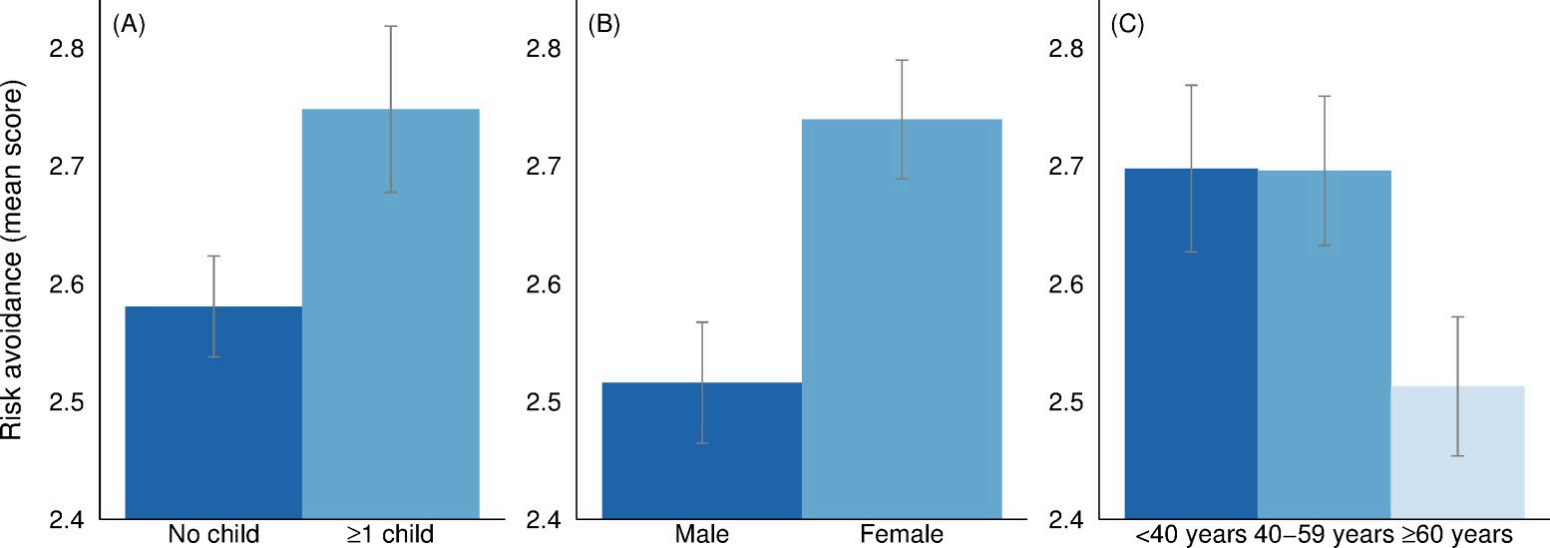
Levels of preference for radiation risk avoidance by family situation, gender, and age. Confidence intervals are displayed at the 95% level.

Fig 2 displays the mean score of preference for risk avoidance by family situation, gender, and age. The results are largely in line with the non-parametric results on worry for radiation exposure. Respondents in households with at least one child are more inclined to leave a neighborhood that has been affected by nuclear fallout even though the radiation levels have been declared safe. In the four-point Likert scale, respondents in households without children report an average score of 2.58 (2.53–2.62) while respondents with at least one child report an average of 2.75 (2.68–2.82). The difference is highly statistically significant (MW p-value = 0.000). Gender is important also here and female respondents report a mean score of 2.74 (2.69–2.79) while male respondents report a mean score of 2.52 (2.46–2.57); also this difference is highly statistically significant (MW p-value = 0.000). In regards to age, there is no statistically significant difference between the two youngest age groups (respondents under 40 years old and between 40 and 59 years old). However, respondents over 60 years old report a weaker preference for risk avoidance than the other two age groups (MW p-value = 0.000). A Pearson’s chi-squared test gives an overall p-value of 0.000 –giving some indication that there is an overall negative relationship between age and the preference to move away from a decontaminated fallout area.

The correlation between the two response variables is 0.57. Spearman's rank correlation is also 0.57.

### Regression analysis

Table 2 shows the relationship between worry with respect to radiation exposure and our demographic variables of interest using the ordered logistic regression technique. The univariate results from Model 1 are largely consistent with the non-parametric results presented above. Family situation seems to have a substantial effect and display high coefficients across models; having one or more children in the household clearly increase the probability of being anxious over radiation exposure. The effect is even larger when it comes to gender. Being female substantially increases the probability of a higher degree of concern over radiation exposure. Also, this effect is highly statistically significant across models. Being 60 years old or more, as seen above in the non-parametric testing, is shown also here to decrease the probability of high levels of worry over radiation exposure. However, this effect loses its statistical significance in Model 2, where we adjust for income, educational level, the distance to the nearest nuclear power plant, geographical sample group, and if the respondent is a member of any local organization. In Model 3, the coefficients are approaching zero and are far from being statistically significant, suggesting that the age effect on worry for radiation exposure goes through some of the other variables included in the model e.g. income or family situation.

**Table 2:** Ordered logit model for worry for radiation exposure; the effect of family situation, gender, and age.

	Model 1*		Model 2**		Model 3***	
	OR (95% CI)	p-value	aOR (95%CI)	p-value	aOR (95%CI)	p-value
*Family situation*						
≥1 child in household	0.29 (0.11–0.47)	0.002	0.39 (0.18–0.61)	0.000	0.31 (0.07–0.56)	0.012
*Gender*						
Female	0.94 (0.78–1.09)	0.000	0.90 (0.72–1.08)	0.000	0.88 (0.69–1.07)	0.000
*Age*						
<40 years	Ref.		Ref.		Ref.	
40–59 years	-0.04 (-0.25–0.18)	0.741	0.08 (-0.18–0.33)	0.566	0.01 (-0.25–0.27)	0.958
≥60 years	-0.25 (-0.45–0.05)	0.013	-0.18 (-0.44–0.08)	0.171	-0.06 (-0.33–0.22)	0.684
Pseudo-R^2^ (average)	(0.008)		(0.016)		0.029	
VIF score (average)			(2.52)		2.46	
N (average)	(2149)		(1678)		1626	

*Model 1: Univariate model

**Model 2: Control variables included (the variables presented were separately included)

***Model 3: All variables included

The 95% level confidence intervals and p-values are computed using heteroscedasticity-consistent standard errors

Table 3 shows the ordered logistic results for preferences for avoiding radiation exposure by moving. In regards to family situation, Model 1 and Model 2 echoes the effect from the non-parametric analysis of having one or more children in the household on levels of preference for radiation risk avoidance. However, in Model 3, the coefficient loses its statistical significance, indicating that the effect goes through age and gender. The results furthermore clearly show that females are more likely to avoid radiation exposure with statistically significant coefficients across models. The results also make clear with statistically significant results across models that people that are 60 years old or above are less likely to move than the younger age groups.

**Table 3:** Ordered logit model for levels of preference for risk avoidance; the effect of family situation, gender, and age.

	Model 1*		Model 2**		Model 3***	
	OR (95% CI)	p-value	aOR (95%CI)	p-value	aOR (95%CI)	p-value
*Family situation*						
≥1 child in household	0.37 (0.19–0.55)	0.000	0.28 (0.07–0.50)	0.011	0.18 (-0.07–0.43)	0.153
*Gender*						
Female	0.48 (0.32–0.63)	0.000	0.47 (0.28–0.65)	0.000	0.48 (0.29–0.67)	0.000
*Age*						
<40 years	Ref.		Ref.		Ref.	
40–59 years	0.02 (-0.19–0.22)	0.883	-0.02 (-0.26–0.23)	0.888	-0.06 (-0.31–0.18)	0.612
≥60 years	-0.39 (-0.58–0.19)	0.000	-0.34 (-0.59–0.09)	0.008	-0.27 (-0.53–0.02)	0.038
Pseudo-R^2^ (average)	(0.005)		(0.010)		0.016	
VIF score (average)			(2.51)		2.46	
N (average)	(2138)		(1674)		1622	

*Model 1: Univariate model

**Model 2: Control variables included (the variables presented were separately included)

***Model 3: All variables included

The 95% level confidence intervals and p-values are computed using heteroscedasticity-consistent standard errors

### Robustness tests

As robustness tests, we re-estimated the regression models using two other estimators. First, we estimated a logit model using binary versions of the dependent variables that indicate if the respondent has opted for any of the two last ordered options of the respective Likert scale (S2 Table). Second, we treat the dependent variables as continuous and run the regression models again, but now with an ordinary least squares estimator (S3 Table). Both estimators display very similar results as the ordered logistic estimator. The only real exception being the coefficients for the oldest age group that loses some statistical significance in Model 2 and Model 3 in the logit model. However, the ordinary least square estimator instead strengthens the statistical significance in Model 2 and Model 3. Both the logit and ordinary least squares estimators display higher (Pseudo-)R-squared measures than the ordered logit estimator.

In addition, since the question formulation for one of the response variable questions indicate that it is the government that has declared the radiation levels to be safe, we also re-estimated all models while holding the reported trust in the government constant. We constructed the additional control variables from a question in the survey where the respondents were asked to rate to what extent they believe the government agencies are able to communicate correct and objective information about the sanitation on a five-ordered Likert scale. The results are shown to be close to identical as before including these additional controls while they increase the Pseudo-R-squared measure (S4 Table).

## Discussion

The purpose of this article was to study the association between gender and life-stage factors and levels of both worry for ionizing radiation exposure and preference for risk avoidance. It was prompted by research suggesting that societies’ preparedness for nuclear accidents could be improved if governing bodies gain a better understanding of how citizens’ risk perception and behavior vary by demographics [8, 15] and by the fact that evacuees do not seem to move back to decontaminated areas to the extent that government authorities expect [6, 8, 9]. To pursue this aim, a scenario-based survey answered by 2,291 adult Swedes was completed and analyzed. Overall, our results reveal a majority expressing quite high-to-high levels of both worry for radiation exposure and preference for risk avoidance. In the following, we will summarize the results in relation to previous research as well as offer some takeaways for future research as well as risk management practice.

The first major finding of this research is that gender is an important and highly statistically significant determinant of worry for radiation exposure. The results also show that women are more likely to express the preference not to stay in an area that has been decontaminated after nuclear fallout. This result is also statistically significant. Thus, first, the study shows similar results regarding gender, risk and behavioral attitudes in relation to radiation and home location as research completed in Japan after the Fukushima Daiichi accident [6, 8, 9]. In culturally and geographically diverse countries such as Sweden and Japan, women perceive radiation risk to be higher than men do and are comparatively less likely to stay in a contaminated but sanitized area. Thus, we also demonstrate completely different results than Olofsson and Rashid [17] who argued that perceptions of risk in a quite gender-equal context like Sweden will show significant differences along variables counting for social security but not for gender. By contrast, we demonstrated that gender differences are statistically significant across regression models that control for social security characteristics like income, education level and community organization membership.

The second major finding is that while we do not find any clear evidence that people who are 60 years old and above differ from the other respondents in terms of worry for radiation exposure, we find strong evidence that they are more likely to stay in a decontaminated fallout area. These results are in line with research after the Fukushima Daiichi accident, which has found that respondents 65 years and older demonstrate significantly less worry about ionizing radiation than younger groups [44] and regarding behavioral attitudes toward home location, a significantly larger proportion of older evacuees returned to their homes in decontaminated areas than younger groups did [9]. These results may also pose implications for risk management.

The third major finding is that citizens’ family situation matter for their level of worry for radiation exposure and their reported inclination to leave a neighborhood that has been affected by nuclear fallout. Our study thus provides further evidence for the relationship between parenthood and high levels of worry for radiation risk as well as comparatively greater propensity to leave affected areas [6, 8, 9] and thus a stronger preference for risk avoidance [36]. The results may also speak to a broader research agenda on demographics and risk proneness and risk aversion, showing parents’ propensity for altruistic choices and risk avoidance [20, 45, 46, 47, 48, 49].

Rather than overly emotional, we might see women and parents as quite right in their perception of higher risk in light of new data showing that women and young children are actually subjected to higher risk of cancer than others, at exposure to the same ground deposition level of Cs-137, and to such a degree that the researchers suggest that current risk models should be revised [12]. While the above results are interesting in themselves, they also offer some takeaways for risk-management practice. Since risk management regarding nuclear and radiation accidents has so far largely focused on evacuees returning to and continuing to live in their homes after restoration [1], our study provokes consideration of alternatives. Fifty-eight percent express fairly or very strong worry about radiation exposure in a scenario where the authorities claim that radiation levels are harmless. Responding to the same scenario, 56.5 percent either express that it is not very likely, or not at all likely, that they would continue living in the area (see Table 1). A majority expresses both high levels of worry and preference for risk avoidance. Thus, there appears to be a discrepancy between the authorities’ typical, planned measures in the event of a nuclear and radiation accident and a majority of the public’s preferences. Notably, the risk management literature suggests that acceptable and tolerated risks can be managed with risk mitigation measures, without creating long-standing conflicts, whereas risks that are not tolerated rather necessitate protection by disconnecting risk bearer and risk sources if the risk cannot be extinguished [24]. Consequently, remediation and relocation, which is a measure that does not eliminate but reduce the risk, often with remaining high-risk spots, would be adequate to implement as the only solution in case elevated ionizing radiation exposure was tolerated and an evenly distributed risk. Since it is not, but rather a sharply stigmatized risk with higher LAR of cancer for women and the lower the age group [12], one can debate whether it is rational in all cases to invest hugely in remediation that is unlikely to lead to the degree of risk reduction that is adequate for non-tolerated risks, and whether to consider allocating resources to allow a set of options that are accepted by and pose lower health risks to larger sections of the population. What should also be taken into account in this discussion, is that especially many among those expressing high levels of worry and preference for risk avoidance are men and women in economically productive age groups, women of childbearing age and parents with children in their household. Problems related to demographics, which are described as a major challenge in the Western world and Japan, with aging populations and low childbirth rates could likely be particularly pronounced in the decontaminated area and hold back its regrowth and productivity. However, these are consequences that our results indicate and future research could determine.

Next, a few limitations should be taken into account when interpreting the findings from this study. To begin with, although we together with field-experienced radiophysicists constructed an as realistic as possible scenario, we cannot exclude the possibility that the respondents’ risk attitudes are to some extent different than real-life reactions. A ‘hypothetical bias’ can have an impact on reported decisions on whether to move from a remediation area, in that some respondents may underestimate financial constraints and ramifications that affect decisions in real life. To mitigate this potential bias, the survey included questions about the impact of economic factors on housing decisions and household finances. The respondents were thus reminded of some of the practical limitations of everyday life. In addition, the Pseudo-R-squared measures are low in both Table 2 and Table 3, showing that our regressors explain at most 2.9 percent of the variation in the dependent variable. Low levels of the R-squared measure are however expected in this kind of study, considering that it concerns human behavior in a hypothetical scenario. Naturally, many different factors come into play when making decisions in a scenario like ours that simply are not possible to control for. In addition, it should be stressed that the contribution of our estimates does not primarily lay in their precision, but rather the association between the variables of interest and the response variables.

Finally, we propose, first, that future research investigate more closely how demographically distorted a recovery area is likely to become, as well as the economic and infrastructural consequences of this. Second, further research should identify the cost of an alternative risk management path that involves helping people financially to start over in communities that have not suffered human-induced radiation pollution. Third, we want to see further research on the importance of variables other than gender and life-stage factors on public risk response, such as respondents’ geographical proximity to nuclear power plants as well as respondents’ socioeconomics conditions.

## Conclusions

Building on results of a scenario-based survey study (N 2,291), this article proposes and examines associations between gender, life-stage factors and public risk response in a nuclear fallout scenario. Six hypotheses were largely confirmed. To begin with, women express both statistically significant higher levels of worry for the risk of ionizing radiation exposure and preference for risk avoidance than men do (H1 and H2). Moreover, two age groups (20–39 and 40 to 59 years old) show some signs of higher levels of worry for the risk of ionizing radiation exposure and significantly higher preference for risk avoidance than respondents aged 60 years and older do (H3 and H4). Lastly, parents with children lin the home express both statistically significant higher levels of worry for the risk of ionizing radiation exposure and preference for risk avoidance than non-parents do (H5 and H6). The study contributes to risk research by showing that the tendency observed in Japan–that women, parents and younger people express greater concern and a stronger preference for risk avoidance by moving in the aftermath of the Fukushima Daiichi accident [6, 8, 9]–could also be observed in our scenario-based study in Northern Europe. We furthermore observed correlations between levels of worry and the strength of preference for risk avoidance by moving. Moreover, women and parents might be quite right in their perception of higher risk, as new data show higher LAR of cancer for women than for men, and higher risk the lower age group, at exposure to the same ground deposition level of Cs-137 [12]. The study finally demonstrates results contrary to research that has suggested that gender is not a determinant of risk perception [17], in that our results demonstrated statistically significant gender differences across regression models controlling for social security variables. Suggesting some takeaways for risk management, the results imply that, according to the view of the majority, ionizing radiation exposure is largely an intolerable risk. An alternative to relying only on the most common, large-scale risk management strategy of mitigating radiation risk through remediation, would involve allocating resources and social support to broaden the options, including assisting risk bearers in relocating away from the risk source. With that being said, our study was based on a hypothetical scenario that is subject to some uncertainty from possible differences compared to real-life experiences. Our discussion on possible risk management takeaways is therefore based holistically on the statistically significant associations we found between studied variables and the fact that our scenario-based results are in agreement with studies of public responses to real-life ionizing radiation risks in Japan. Proposed avenues for future research include studies of (a) the demographic changes in radiation-polluted areas, implied by our research, and the socio-economic costs involved; (b) the association between both respondents’ proximity to nuclear operations, their socio-economic conditions and risk perception; and (c) the methods and costs of a risk management strategy focusing on long-term relocation.

## Supporting information

S1 Appendix Translated transcript of scenario. (DOCX)

S1 Raw data Survey data. (DTA)

S1 Table (DOCX)

S2 Table (DOCX)

S3 Table (DOCX)

S4 Table (DOCX)
