# Supplementary material for: Gender and life-stage dependent reactions to the risk of radioactive contamination: A survey experiment in Sweden
Source: PLoS One. 2020 Apr 30;15(4):e0232259. doi: 10.1371/journal.pone.0232259 (PMC7192462; doi:10.1371/journal.pone.0232259)
Supplement: S1 Appendix — (DOCX) [file pone.0232259.s001.docx]

**S1 Appendix. Translated Transcript of Scenario.**

A nuclear accident has occurred in Sweden and your residential area is contaminated with radioactive substances. You are evacuated to temporary housing while the authorities are cleaning up parts of your residential area. The remediation includes removal of contaminated land close to the house, as well as cleaning and removal of radioactive material from roof and facade surfaces and, if necessary, also from indoor surfaces. The disposal of adjacent land is likely to damage vegetation such as flowerbeds. The remediation measures can take up to one year to complete.

After the clean-up, measurements of houses and gardens show that the levels of radioactive substances are so low that they are considered harmless. However, there are areas around your residential area that show levels of radiation that are so high that you are not allowed to live there and in some cases they require special permits for access. The authorities advise parents not to allow their children to play freely in surrounding natural areas. They also advise against hunting and berry and mushroom picking. Some industries (especially hunting, fishing, agriculture) may find that selling certain products is prohibited or difficult. The following questions try to find out how you relate to living in such a residential area.
