# Supplementary material for: Gender and life-stage dependent reactions to the risk of radioactive contamination: A survey experiment in Sweden
Source: PLoS One. 2020 Apr 30;15(4):e0232259. doi: 10.1371/journal.pone.0232259 (PMC7192462; doi:10.1371/journal.pone.0232259)
Supplement: S1 Table — (DOCX) [file pone.0232259.s003.docx]

**S3 Table. Descriptive statistics of dataset.**

| **Variable** | **Coding** | **N** | **Mean** | **SD** | **95% CI** | **Min** | **Max** |
| --- | --- | --- | --- | --- | --- | --- | --- |
| Worry for radiation exposure | 1 To a very small extent; 2 To a somewhat small extent; 3 Neither small nor large extent; 4 To a somewhat large extent; 5 To a very large extent | 2,198 | 3.460 | 1.191 | (3.375-3.490) | 1 | 5 |
| Likeliness to stay in a decontaminated area | 1 Very likely; 2 Somewhat likely;  3 Not very likely; 4 Not at all likely | 2,185 | 2.623 | 0.866 | (2.582-2.665) | 1 | 4 |
| Family situation | 0 No child; 1 ≥1 child | 2,148 | 0.277 | 0.448 | (0.276-0.320) | 0 | 1 |
| Gender | 0 Male; 1 Female | 2,245 | 0.482 | 0.500 | (0.451-0.500) | 0 | 1 |
| Age | 1 <40 years; 2 40-59 years;  3 ≥60 years | 2,142 | 2.137 | 0.791 | (2.115-2.191) | 1 | 3 |
| Income | 1 <37,000 SEK; 2 37,000-74,999 SEK; 3 ≥75,000 SEK | 1,963 | 1.929 | 0.748 | (1.910-1.983) | 1 | 3 |
| Education | 1 Upper secondary edu.; 2 Post-secondary edu.; 3 University degree | 2,143 | 2.224 | 0.826 | (2.231-2.310) | 1 | 3 |
| Proximity to nuclear power plant | 1 <20 km; 2 20-50 km; 3 60-100 km; 4 >100 km; 5 Don't know | 2,149 | 3.313 | 0.951 | (3.241-3.331) | 1 | 5 |
| Sample group | 1 Sweden; 2 Uppsala; 3 Kalmar;  4 Halland | 2,245 | 2.013 | 1.154 | (1.933-2.044) | 1 | 4 |
| Member of sports/outdoor association | 0 No; 1 Yes | 1,851 | 0.376 | 0.485 | (0.358-0.405) | 0 | 1 |
| Member of environmental organization | 0 No; 1 Yes | 1,851 | 0.131 | 0.338 | (0.118-0.151) | 0 | 1 |
| Member of political party | 0 No; 1 Yes | 1,851 | 0.189 | 0.392 | (0.170-0.208) | 0 | 1 |
| Member of workers' union | 0 No; 1 Yes | 1,851 | 0.477 | 0.500 | (0.460-0.509) | 0 | 1 |
| Member of cultural association | 0 No; 1 Yes | 1,851 | 0.211 | 0.408 | (0.190-0.229) | 0 | 1 |
