# Supplementary material for: Gender and life-stage dependent reactions to the risk of radioactive contamination: A survey experiment in Sweden
Source: PLoS One. 2020 Apr 30;15(4):e0232259. doi: 10.1371/journal.pone.0232259 (PMC7192462; doi:10.1371/journal.pone.0232259)
Supplement: S2 Table — (DOCX) [file pone.0232259.s004.docx]

**S4 Table. Logit model for (A) worry for radiation exposure and (B) levels of preference for radiation risk avoidance; the effect of family situation, gender, and age.**

| **(A)** | **Model 1*** | | | **Model 2**** | | | **Model 3***** | | |
| --- | --- | --- | --- | --- | --- | --- | --- | --- | --- |
|  | **OR (95% CI)** | **p-value** | | **aOR (95%CI)** | | **p-value** | **aOR (95%CI)** | **p-value** | |
| *Family situation* |  |  | |  | |  |  |  | |
| ≥1 child in household | 0.28 (0.09-0.48) | 0.004 | | 0.37 (0.14-0.61) | | 0.002 | 0.34 (0.07-0.61) | 0.014 | |
| *Gender* |  |  | |  | |  |  |  | |
| Female | 0.82 (0.65-1.00) | 0.000 | | 0.76 (0.56-0.97) | | 0.000 | 0.74 (0.53-0.95) | 0.000 | |
| *Age* |  |  | |  | |  |  |  | |
| <40 years | Ref. |  | | Ref. | |  | Ref. |  | |
| 40-59 years | 0.01 (-0.22-0.24) | 0.946 | | 0.09 (-0.18-0.37) | | 0.515 | 0.03 (-0.25-0.32) | 0.827 | |
| ≥ 60 years | -0.21 (-0.43-0.01) | 0.060 | | -0.16 (-0.45-0.12) | | 0.267 | -0.04 (-0.35-0.27) | 0.793 | |
|  |  |  | |  | |  |  |  | |
| Pseudo-R^2^ (average) | (0.011) |  | | (0.024) | |  | 0.042 |  | |
| VIF score (average) |  |  | | (2.52) | |  | 2.46 |  | |
| N (average) | (2149) |  | | (1678) | |  | 1626 |  | |
| *Model 1: Univariate model | |  | |  | |  |  |  | |
| **Model 2: Control variables included (the variables presented were separately included) | | | | | | | | |  |
| ***Model 3: All variables included | |  | |  | |  |  |  | |
| The response variable is 0 if respondent answered “To a very small extent”, “To a somewhat small extent”, or “Neither small nor large extent”. The response variable is 1 if the respondent answered “To a somewhat large extent” or “To a very large extent”. The 95% level confidence intervals and p-values are computed using heteroscedasticity-consistent standard errors | | | | | | | | | |
|  | | | | | | | | | |
| **(B)** | **Model 1*** | | | **Model 2**** | | | **Model 3***** | | |
|  | **OR (95% CI)** | | **p-value** | **aOR (95%CI)** | **p-value** | | **aOR (95%CI)** | **p-value** | |
| *Family situation* |  | |  |  |  | |  |  | |
| ≥1 child in household | 0.38 (0.19-0.58) | | 0.000 | 0.30 (0.06-0.53) | 0.013 | | 0.21 (-0.06-0.47) | 0.124 | |
| *Gender* |  | |  |  |  | |  |  | |
| Female | 0.50 (0.32-0.67) | | 0.000 | 0.50 (0.30-0.71) | 0.000 | | 0.52 (0.31-0.73) | 0.000 | |
| *Age* |  | |  |  |  | |  |  | |
| <40 years | Ref. | |  | Ref. |  | | Ref. |  | |
| 40-59 years | -0.00 (-0.23-0.23) | | 0.987 | -0.04 (-0.31-0.24) | 0.789 | | -0.08 (-0.36-0.20) | 0.558 | |
| ≥60 years | -0.40 (-0.62--0.18) | | 0.000 | -0.27 (-0.56-0.01) | 0.061 | | -0.20 (-0.51-0.11) | 0.199 | |
|  |  | |  |  |  | |  |  | |
| Pseudo-R^2^ (average) | (0.008) | |  | (0.015) |  | | 0.026 |  | |
| VIF score (average) |  | |  | (2.51) |  | | 2.46 |  | |
| N (average) | (2138) | |  | (1674) |  | | 1622 |  | |
| *Model 1: Univariate model | | |  |  |  | |  |  | |
| **Model 2: Control variables included (the variables presented were separately included) | | | | | | | | |  |
| ***Model 3: All variables included | | |  |  |  | |  |  | |
| The response variable is 0 if respondent answered “Very likely” or “Somewhat likely” and 1 if respondent answered “Not very likely” or “Not at all likely”. The 95% level confidence intervals and p-values are computed using heteroscedasticity-consistent standard errors | | | | | | | | | |
