# Supplementary material for: Gender and life-stage dependent reactions to the risk of radioactive contamination: A survey experiment in Sweden
Source: PLoS One. 2020 Apr 30;15(4):e0232259. doi: 10.1371/journal.pone.0232259 (PMC7192462; doi:10.1371/journal.pone.0232259)
Supplement: S3 Table — (DOCX) [file pone.0232259.s005.docx]

**S5 Table. Ordinary least square regression for (A) worry for radiation exposure and (B) levels of preference for radiation risk avoidance; the effect of family situation, gender, and age.**

| **(A)** | **Model 1*** | | **Model 2**** | | **Model 3***** | |
| --- | --- | --- | --- | --- | --- | --- |
|  | **β (95% CI)** | **p-value** | **β (95%CI)** | **p-value** | **β (95%CI)** | **p-value** |
| *Family situation* |  |  |  |  |  |  |
| ≥1 child in household | 0.15 (0.03-0.27) | 0.012 | 0.22 (0.09-0.36) | 0.001 | 0.17 (0.03-0.32) | 0.021 |
| *Gender* |  |  |  |  |  |  |
| Female | 0.59 (0.49-0.68) | 0.000 | 0.55 (0.44-0.66) | 0.000 | 0.54 (0.42-0.65) | 0.000 |
| *Age* |  |  |  |  |  |  |
| <40 years | Ref. |  | Ref. |  | Ref. |  |
| 40-59 years | -0.02 (-0.16-0.11) | 0.735 | 0.06 (-0.10-0.22) | 0.480 | 0.02 (-0.14-0.17) | 0.844 |
| ≥60 years | -0.14 (-0.27--0.01) | 0.030 | -0.10 (-0.26-0.07) | 0.255 | -0.03 (-0.20-0.14) | 0.717 |
|  |  |  |  |  |  |  |
| R^2^ (average) | (0.022) |  | (0.044) |  | 0.078 |  |
| VIF score (average) |  |  | (2.52) |  | 2.46 |  |
| N (average) | (2149) |  | (1678) |  | 1626 |  |
| *Model 1: Univariate model | |  |  |  |  |  |
| **Model 2: Control variables included (the variables presented were separately included) | | | | | |  |
| ***Model 3: All variables included | |  |  |  |  |  |
| The 95% level confidence intervals and p-values are computed using heteroscedasticity-consistent standard errors | | | | | | |
|  | | | | | | |
| **(B)** | **Model 1*** | | **Model 2**** | | **Model 3***** | |
|  | **β (95% CI)** | **p-value** | **β (95%CI)** | **p-value** | **β (95%CI)** | **p-value** |
| *Family situation* |  |  |  |  |  |  |
| ≥1 child in household | 0.17 (0.08-0.25) | 0.000 | 0.13 (0.03-0.23) | 0.010 | 0.08 (-0.03-0.19) | 0.162 |
| *Gender* |  |  |  |  |  |  |
| Female | 0.22 (0.15-0.30) | 0.000 | 0.21 (0.12-0.29) | 0.000 | 0.21 (0.13-0.30) | 0.000 |
| *Age* |  |  |  |  |  |  |
| <40 years | Ref. |  | Ref. |  | Ref. |  |
| 40-59 years | -0.00 (-0.10-0.09) | 0.971 | -0.01 (-0.13-0.10) | 0.823 | -0.03 (-0.14-0.08) | 0.602 |
| ≥60 years | -0.18 (-0.28--0.09) | 0.000 | -0.16 (-0.28--0.05) | 0.006 | -0.13 (-0.25--0.01) | 0.029 |
|  |  |  |  |  |  |  |
| R^2^ (average) | (0.012) |  | (0.027) |  | 0.042 |  |
| VIF score (average) |  |  | (2.51) |  | 2.46 |  |
| N (average) | (2138) |  | (1674) |  | 1622 |  |
| *Model 1: Univariate model | |  |  |  |  |  |
| **Model 2: Control variables included (the variables presented were separately included) | | | | | |  |
| ***Model 3: All variables included | |  |  |  |  |  |
| The 95% level confidence intervals and p-values are computed using heteroscedasticity-consistent standard errors | | | | | | |
