# Supplementary material for: Gender and life-stage dependent reactions to the risk of radioactive contamination: A survey experiment in Sweden
Source: PLoS One. 2020 Apr 30;15(4):e0232259. doi: 10.1371/journal.pone.0232259 (PMC7192462; doi:10.1371/journal.pone.0232259)
Supplement: S4 Table — (DOCX) [file pone.0232259.s006.docx]

**S6 Table. Ordered logit model for (A) worry for radiation exposure and (B) levels of preference for radiation risk avoidance; the effect of family situation, gender, and age when controlling for government trust.**

| **(A)** | **Model 1*** | | **Model 2**** | | **Model 3***** | |
| --- | --- | --- | --- | --- | --- | --- |
|  | **OR (95% CI)** | **p-value** | **aOR (95%CI)** | **p-value** | **aOR (95%CI)** | **p-value** |
| *Family situation* |  |  |  |  |  |  |
| ≥1 child in household | 0.29 (0.11-0.47) | 0.002 | 0.37 (0.15-0.58) | 0.001 | 0.28 (0.03-0.53) | 0.028 |
| *Gender* |  |  |  |  |  |  |
| Female | 0.94 (0.78-1.09) | 0.000 | 0.96 (0.78-1.15) | 0.000 | 0.95 (0.77-1.14) | 0.000 |
| *Age* |  |  |  |  |  |  |
| <40 years | Ref. |  | Ref. |  | Ref. |  |
| 40-59 years | -0.04 (-0.25-0.18) | 0.741 | 0.03 (-0.23-0.29) | 0.812 | -0.04 (-0.31-0.22) | 0.754 |
| ≥60 years | -0.25 (-0.45--0.05) | 0.013 | -0.21 (-0.47-0.05) | 0.117 | -0.10 (-0.37-0.18) | 0.481 |
|  |  |  |  |  |  |  |
| Pseudo-R^2^ (average) | (0.008) |  | (0.030) |  | 0.045 |  |
| VIF score (average) |  |  | (2.29) |  | 2.27 |  |
| N (average) | (2149) |  | (1676) |  | 1624 |  |
| *Model 1: Univariate model | |  |  |  |  |  |
| **Model 2: Control variables and government trust variable included (the variables presented were separately included) | | | | | | |
| ***Model 3: All variables including government trust variable included | | | | |  |  |
| The 95% level confidence intervals and p-values are computed using heteroscedasticity-consistent standard errors | | | | | | |
|  | | | | | | |
| **(B)** | **Model 1*** | | **Model 2**** | | **Model 3***** | |
|  | **OR (95% CI)** | **p-value** | **aOR (95%CI)** | **p-value** | **aOR (95%CI)** | **p-value** |
| *Family situation* |  |  |  |  |  |  |
| ≥1 child in household | 0.37 (0.19-0.55) | 0.000 | 0.27 (0.05-0.49) | 0.016 | 0.17 (-0.08-0.42) | 0.190 |
| *Gender* |  |  |  |  |  |  |
| Female | 0.48 (0.32-0.63) | 0.000 | 0.53 (0.34-0.71) | 0.000 | 0.55 (0.36-0.74) | 0.000 |
| *Age* |  |  |  |  |  |  |
| <40 years | Ref. |  | Ref. |  | Ref. |  |
| 40-59 years | 0.02 (-0.19-0.22) | 0.883 | -0.08 (-0.32-0.17) | 0.546 | -0.13 (-0.38-0.12) | 0.321 |
| ≥60 years | -0.39 (-0.58--0.19) | 0.000 | -0.38 (-0.63--0.12) | 0.004 | -0.32 (-0.58--0.05) | 0.019 |
|  |  |  |  |  |  |  |
| Pseudo-R^2^ (average) | (0.005) |  | (0.031) |  | 0.037 |  |
| VIF score (average) |  |  | (2.29) |  | 2.27 |  |
| N (average) | (2138) |  | (1672) |  | 1620 |  |
| *Model 1: Univariate model | |  |  |  |  |  |
| **Model 2: Control variables and government trust variable included (the variables presented were separately included) | | | | | | |
| ***Model 3: All variables including government trust variable included | | | | |  |  |
| The 95% level confidence intervals and p-values are computed using heteroscedasticity-consistent standard errors | | | | | | |
